# Unraveling the causality between chronic obstructive pulmonary disease and its common comorbidities using bidirectional Mendelian randomization

**DOI:** 10.1186/s40001-024-01686-x

**Published:** 2024-02-26

**Authors:** Zihan Wang, Yongchang Sun

**Affiliations:** Department of Respiratory and Critical Care Medicine, Peking University Third Hospital; Research Center for Chronic Airway Diseases, Peking University Health Science Center, Beijing, China

**Keywords:** Mendelian randomization, Single nucleotide polymorphisms, COPD, Comorbidity, Causality

## Abstract

**Background:**

Chronic obstructive pulmonary disease (COPD) frequently coexists with various diseases, yet the causal relationship between COPD and these comorbidities remains ambiguous. As a result, the aim of our study is to elucidate the potential causality between COPD and its common comorbidities.

**Methods:**

We employed the Mendelian randomization (MR) method to analyze single nucleotide polymorphism (SNP) data of common comorbidities with COPD from FinnGen and Integrative Epidemiology Unit (IEU) databases. Causality was primarily assessed using the inverse variance weighting (IVW) method. Multivariable Mendelian randomization (MVMR) analysis was also conducted to eliminate the interference of smoking-related phenotypes. Sensitivity analysis was conducted to ensure the reliability of our findings.

**Results:**

Preliminary univariable MR revealed an increased risk of lung squamous cell carcinoma (LUSC) (IVW: OR = 1.757, 95% CI = 1.162–2.657, *P* = 0.008), chronic kidney disease (CKD) (IVW: OR = 1.193, 95% CI = 1.072–1.326, *P* < 0.001), chronic periodontitis (IVW: OR = 1.213, 95% CI = 1.038–1.417, *P* = 0.012), and heart failure (HF) (IVW: OR = 1.127, 95% CI = 1.043–1.218, *P* = 0.002). Additionally, the reverse MR analysis indicated that genetic susceptibility to HF (IVW: OR = 1.272, 95% CI = 1.084–1.493, *P* = 0.003), obesity (IVW: OR = 1.128, 95% CI = 1.056–1.205, *P* < 0.001), depression (IVW: OR = 1.491, 95% CI = 1.257–1.770, *P* < 0.001), and sleep apnea syndrome (IVW: OR = 1.209, 95% CI = 1.087–1.345, *P* < 0.001) could raise the risk of COPD. The MVMR analysis showed no causal effect of COPD on susceptibility to chronic periodontitis after adjusting for smoking.

**Conclusions:**

Our study identified that COPD may elevate the risk of LUSC, HF, and CKD. Additionally, our analysis revealed that HF, sleep apnea symptoms, depression, and obesity might also increase the susceptibility to COPD. These findings revealed a potential causal relationship between COPD and several prevalent comorbidities, which may provide new insights for disease early prediction and prevention.

**Supplementary Information:**

The online version contains supplementary material available at 10.1186/s40001-024-01686-x.

## Background

COPD is one of the most prevalent chronic non-communicable diseases worldwide. Despite the availability of well-established treatment options, many of the symptoms of COPD patients remain uncontrolled, and the mortality is still alarmingly high [1]. Meanwhile, with the extension of human life expectancy, the elderly are confronted with a singular disease and a confluence of multiple systemic diseases referred to as multimorbidity. Patients with COPD also often coexist with other intrapulmonary or extrapulmonary clinical disorders [2, 3], potentially attributable to shared risk factors or the influence of systemic inflammation et al. [4, 5], which further increases the physical burden of patients and social medical expenditure [6, 7]. Strikingly, it was estimated that the final cause of death for more than two-thirds of COPD patients is non-respiratory disorders [2].

Due to time and financial resource constraints, the relationship between COPD and its comorbidities has not been adequately explored. Confounding variables such as aging, smoking, and environmental pollution pose challenges in large-scale cohort studies that provide higher levels of evidence for causation. Therefore, a cost-effective research method that minimizes confounding factors should be employed to establish a causal relationship between COPD and its comorbidities. The findings could lead to early prevention and intervention of multimorbidity within the context of COPD, ultimately improving patients’ quality of life, prolonging their lifespan, and reducing the burden on public health.

The MR analysis is an increasingly employed approach for investigating the causal relationship between observational risk factors and outcomes. Genetic variations, randomly assigned to individuals at birth, are utilized as instrumental variables (IVs) to maximally mitigate the impact of confounding factors mentioned above and reverse causation [8]. Previous studies have utilized MR methods to investigate the causal relationship between COPD and a few comorbidities, including osteoporosis [9], obesity [10], gastroesophageal reflux disease (GERD) [11], and iron deficiency anemia [12]. However, the bidirectional MR analysis has not yet been employed to thoroughly investigate the causal relationship between COPD and its remaining common comorbidities. Therefore, a comprehensive bidirectional MR analysis utilizing up-to-date data is warranted to explore the causal association between COPD and its commonly observed comorbidities.

## Methods

### Study design

**Fig. 1:**
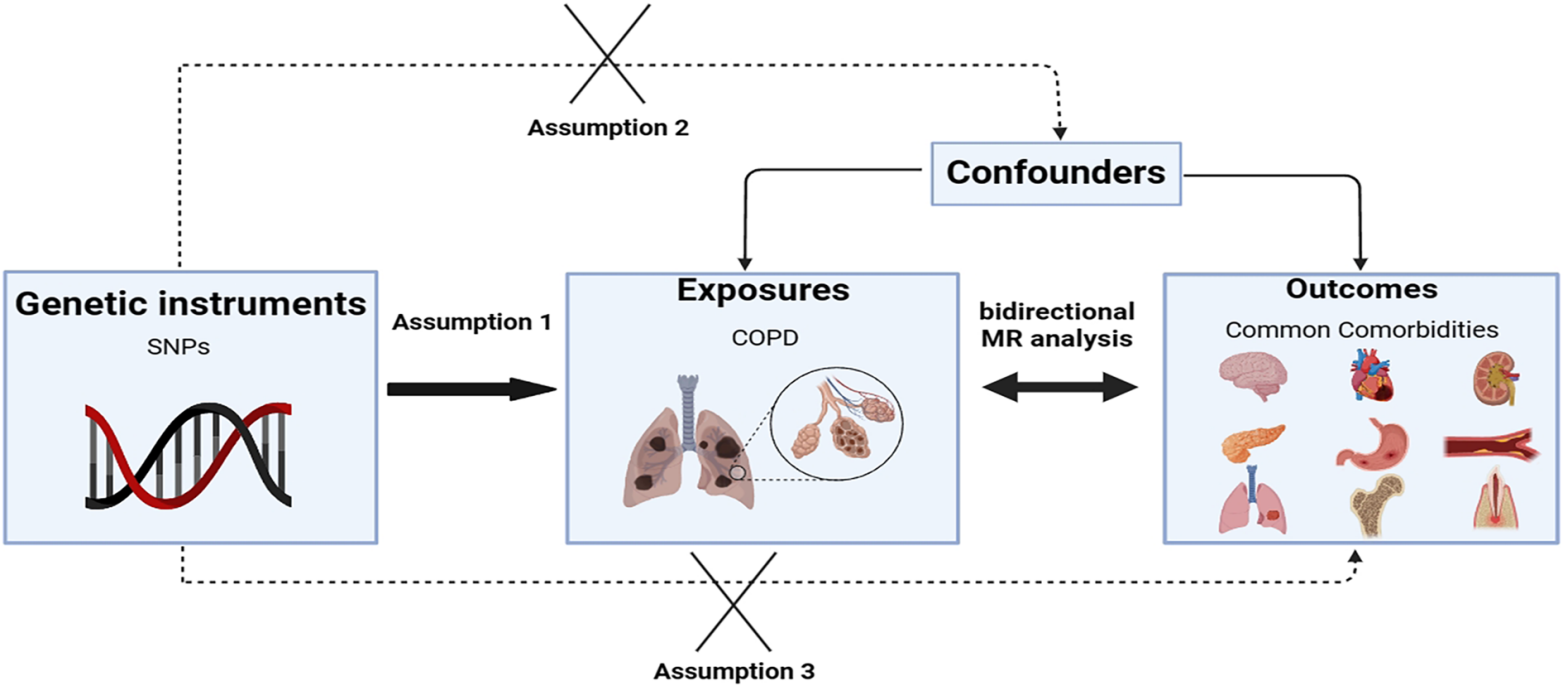
Overall design of our study

This study is reported in accordance with the STROBE-MR guidelines (Additional file 1: Table S1). A schematic overview of the study design is presented in Fig. 1. In brief, we conducted a MR analysis using publicly available summary statistics from FinnGen and IEU datasets. Both exposure and outcome cohorts were limited to individuals of European ancestry to minimize bias arising from population stratification.

## Data source for exposure and outcome

The GWAS summary data on COPD were obtained from the FinnGen Research public database, comprising 18,266 cases and 311,286 controls. Additionally, the GWAS dataset from FinnGen and IEU (https://gwas.mrcieu.ac.uk/) was utilized to derive information on the 24 common comorbidities associated with COPD. In the FinnGen study, diseases were defined using the International Classification of Disease (ICD) code. Specific information about related diseases is shown in Table 1.

**Table 1:** Details of studies included in the MR analyses for the association between COPD and its common comorbidities

Disease	Database	Year	Population	Sample size		Case definition	P-values used to screen IVs
				Cases	Controls		
Chronic obstructive pulmonary disease (COPD)	FinnGen-9	2023	European	18,266	311,286	ICD-10: J43;J44	5 × 10–8
Lung adenocarcinoma	FinnGen-9	2023	European	1,553	287,137	ICD-10: C34	5 × 10–6
Lung squamous carcinoma (LUSC)	FinnGen-9	2023	European	1,413	287,137	ICD-10: C34	5 × 10–6
Small cell lung cancer (SCLC)	FinnGen-9	2023	European	676	287,137	ICD-10: C34	5 × 10–6
Coronary atherosclerosis	FinnGen-9	2023	European	47,550	313,400	ICD-10: I24; I25;T82.2;Z95.11	5 × 10–8
Cerebral atherosclerosis	FinnGen-9	2023	European	322	376,955	ICD-10: I67.2	5 × 10–6
Atrial fibrillation	ebi-a-GCST006414	2018	European	60,620	970,216	Doctor diagnosed/Self report	5 × 10–8
Ischemic heart disease (IHD)	FinnGen-7	2021	European	49,030	260,124	ICD-10: I20-I25	5 × 10–8
Heart failure (HF)	FinnGen-9	2023	European	27,304	349,973	ICD-10: I11.0, I13.0, I13.2, I50	5 × 10–8
Hypertension	FinnGen-9	2023	European	111,581	265,626	ICD-10: I10-I15, I67.4	5 × 10–8
Gastroesophageal reflux	FinnGen-9	2023	European	26,184	320,387	ICD-10: K21	5 × 10–7
Gastric ulcer	FinnGen-9	2023	European	5,935	320,387	ICD-10: K25	5 × 10–7
Duodenal ulcer	FinnGen-9	2023	European	3,520	320,387	ICD-10: K26	5 × 10–8
Venous thromboembolism (VTE)	FinnGen-9	2023	European	19,372	357,905	ICD-10: I26; I80; O87.1; O88.2	5 × 10–8
Obesity	FinnGen-9	2023	European	21,375	355,786	ICD-10: E66	5 × 10–8
Pulmonary embolism (PE)	FinnGen-9	2023	European	9,243	367,108	ICD-10: I26	5 × 10–7
Stroke	FinnGen-9	2023	European	25,398	339,920	ICD-10: I9_SAH; I9_ICH; I9_OTHINTRACRA; I9_STR_EXH; I9_STR_SAH; I9_TIA	5 × 10–8
Osteoporosis	FinnGen-9	2023	European	7,300	358,014	ICD-10: M80, M81, M82	5 × 10–8
Chronic periodontitis	FinnGen-9	2023	European	4,434	259,234	ICD-10: K05.30, K05.31	5 × 10–7
Anxiety	FinnGen-9	2023	European	24,662	337,577	ICD-10: F41.2, F41.3, F41.8, F41.9	5 × 10–7
Depression	FinnGen-9	2023	European	43,280	329,192	ICD-10: F32, F33	5 × 10–8
Anemias	FinnGen-9	2023	European	27,371	88,536	ICD-10: D3	5 × 10–8
Chronic kidney disease (CKD)	FinnGen-9	2023	European	9,073	363,177	ICD-10: N18	5 × 10–7
Sleep apnea syndrome	FinnGen-9	2023	European	38,998	336,659	ICD-10: G47.3	5 × 10–8
Type 2 diabetes	ebi-a-GCST006867	2018	European	61,714	1,178	Doctor diagnosed/Self report	5 × 10–8

## Data source for smoking-related phenotypes

The variables chosen for MVMR analysis to represent smoking exposure were the lifetime smoking index and age of smoking initiation. Wootton et al. developed the comprehensive lifetime smoking index, which incorporates information on smoking intensity, duration, as well as initiation and cessation patterns [13]. Besides, the GWAS summary data on age of smoking initiation (id: ieu-b-24) were retrieved from IEU database.

## Genetic instrumental variables selection

The fundamental requirements for IVs to fulfill the MR assumptions in this research are as follows: 1) The IVs must be associated with the exposure; 2) the IVs must not be associated with any confounders of the exposure-outcome association; and 3) the IVs should not influence the outcome, except possibly via its association with the exposure [14]. To fulfill the assumption 1 of MR, we exclusively included individuals of European ancestry in our study population and, whenever feasible, restricted the *P* value of the selected SNPs to 5 × 10^–8^; however, in cases where there were insufficient IVs for inclusion in the analysis, we could lower the threshold to 5 × 10^–6^ step by step [15–17] (Table 1). To ensure each SNP’s independence, we applied a stringent linkage disequilibrium (LD) correlation coefficient threshold of R^2^ < 0.001 and a clumping window width of 10000 kb [17]. R^2^ for each instrument variant: R^2^ = 2 × EAF × (1 − EAF) × β^2^, where EAF is the effect allele frequency [18] (Additional file 1: Table S2). Furthermore, we excluded SNPs, rs8040868 and rs16969968 that were associated with confounders or outcomes according to the comprehensive retrieved results of the Phenoscanner V2 database and GWAS catalog to fulfill the assumption 2 and 3 [19, 20]. Detailed traits of comprehensive SNPs retrieved in the Phenoscanner V2 database and GWAS catalog was shown in Additional file 1: Table S3. Additionally, to mitigate the impact of weak IVs on the experimental results, SNPs with an F statistic value less than ten were excluded based on the formula *F* = β^2^_exposure_/SE^2^_exposure_. To further verify our findings, we re-performed MR analysis using the threshold of *R*^2^ = 0.01 to select diverse eligible IVs [21].

## Statistical analysis

### UVMR and MVMR analysis

We employed UVMR to evaluate the causal relationship between COPD and its common comorbidities. Given the strong correlation between smoking and COPD, we also conducted the MVMR analysis to assess the independent causal effect of COPD on its comorbidities while adjusting for smoking, which itself has a causal impact on these comorbidities.

The primary approach for MR analysis in UVMR was the inverse-variance weighted (IVW) method, which is utilized to integrate the Wald ratio evaluations of each instrumental variable into a meta-analysis, and is equivalent to performing a weighted linear regression of the associations between the instrumental variables [22]. We also employed various other MR models such as MR-Egger regression, weighted median, and MR pleiotropy residual sum and outlier (MR-PRESSO) methods, were used to examine the aforementioned causality. The MR-Egger regression can be used even when all SNPs are invalid. It estimates the causal effect through a weighted linear regression of gene-outcome coefficients on gene-exposure coefficients [23]. The weighted median approach yields consistent effect estimates when at least half of the weighted variance attributable to horizontal pleiotropy is valid [24]. The MR-PRESSO method, which enables the detection and adjustment for horizontal pleiotropy through outlier removal, was employed to assess pleiotropy [25]. In cases where significant pleiotropy was observed, we utilized the MR-PRESSO method to eliminate outlier SNPs and reanalyze the data. Among these methods, the results obtained from IVW are more reliable compared to the other three approaches. For MVMR analysis, we utilized multivariable IVW (MV-IVW) as the main analysis and multivariable MR-Egger methods as the complementary analysis to estimate the effect of confounder factors on the outcome.

### Sensitivity analysis

The sensitivity analysis encompassed assessments of heterogeneity, pleiotropy, and leave-one-out sensitivity tests. Heterogeneity was evaluated using the IVW method and MR-Egger regression, with Cochran's Q-test *P* > 0.05 means the absence of heterogeneity. If there existed heterogeneity, multiplicative random-effect IVW (IVW-MRE) was chosen as the primary MR analysis method [26]. Horizontal pleiotropy was detected through the intercept term of the MR-Egger method [23]. To address horizontal pleiotropy, we employed MR-PRESSO to identify and remove outliers that were corrected for horizontal pleiotropy (*P* < 0.05 for outlier detection), subsequently evaluating differences in estimates before and after outlier correction[25]. Additionally, a leave-one-out sensitivity test was conducted to evaluate the robustness of our MR findings [27]. For MVMR, the MVMR-Egger intercept test was performed to examine the horizontal pleiotropy [23]. Given that the exposure and outcome samples for the MR analysis were almost derived from the same database, it is inevitable to encounter sample overlap. To address this issue, we assessed the extent of sample overlap between exposure and outcome in the Finnish database and subsequently performed online calculations via a dedicated platform to determine Type I errors and potential bias (https://sb452.shinyapps.io/overlap/) [28].

The “TwosampleMR” package (version 0.5.7) of R software (version 4.2.2) was employed to analyze the causal-effect relationship between COPD and its common comorbidities. A conservative Bonferroni-corrected threshold (*P* < 0.05/24, 0.0021, because 24 comorbidities were evaluated for bidirectional analyses) was adopted to address multiple testing. Associations with *P* < 0.0021 were considered as significant evidence**,** while associations with *P* > 0.0021 and *P* < 0.05 were defined as suggestive evidence based on IVW method.

## Results

### Characteristics of included IVs and overall experimental design

Through the IVs screening process, the number of instrumental variables associated with 24 common comorbidities of COPD ranged from 3 to 187. The specific details regarding these instrumental variables can be found in Table 1 and Additional file 1: Table S3. However, given that the majority of samples were sourced from the FinnGen database and there was some degree of overlap between them, we searched the sample overlap rate in the FinnGen database and calculated the corresponding magnitude of biases and Type I error rate using available online tools (Additional file 1: Table S4). The potential bias caused by sample overlap was considered insignificant (bias estimate < 0.005). All SNPs involved exhibited F statistics greater than 20, indicating robust genetic instruments. Figure 1 provides an overview of the experimental design employed in this study.

## Forward univariable MR analysis

**Fig. 2:**
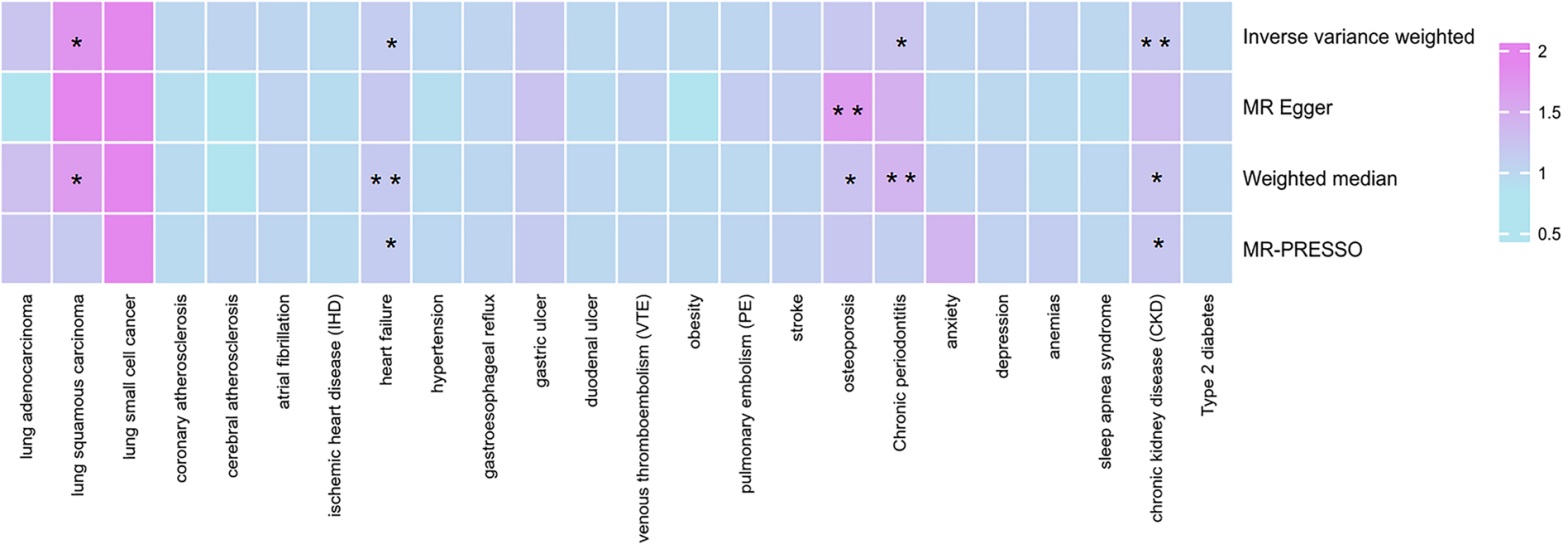
Preliminary UVMR assessments of the associations between genetic susceptibility to COPD and its prevalent comorbidities. Purple boxes denote positive correlations, while light blue boxes signify negative associations. The asterisk “*” represents *P*-values of MR estimates between the Bonferroni-adjusted threshold (*P* < 0.0021) and 0.05. The double asterisk “**” designates *P* < 0.0021

For the preliminary forward MR analysis, it was discovered that COPD is potentially causally related to an increased risk of LUSC (IVW: OR = 1.757, 95% CI = 1.162–2.657, *P* = 0.008), HF (IVW: OR = 1.127, 95% CI = 1.043–1.218, *P* = 0.002), osteoporosis (IVW: OR = 1.197, 95% CI = 1.037–1.381, *P* = 0.014), chronic periodontitis (IVW: OR = 1.213, 95% CI = 1.038–1.417, *P* = 0.012) and CKD (IVW: OR = 1.193, 95% CI = 1.072–1.326, *P* < 0.001) (Fig. 2, Table 2 and Additional file 1: Table S5).

**Table 2:** The preliminary forward UVMR results of significant relationship between COPD and its common comorbidities mainly based on IVW method (*P* < 0.05)

Exposure	Outcome	Method	nSNP	Beta	SE	*P*	OR	95% CI
COPD	Lung squamous carcinoma	IVW	12	0.564	0.211	**0.008**	**1.757**	**1.162–2.657**
		MR-Egger	12	0.690	0.615	0.288	1.993	0.597–6.655
		Weighted median	12	0.519	0.247	**0.036**	1.680	**1.036–2.724**
		MR-PRESSO	12	0.149	0.458	0.752	1.160	0.472–2.849
	Heart failure	IVW	13	0.120	0.040	**0.002**	**1.127**	**1.043–1.218**
		MR-Egger	13	0.161	0.093	0.114	1.174	0.978–1.410
		Weighted median	13	**0.156**	**0.045**	**0.001**	**1.169**	**1.070-1.218**
		MR-PRESSO	13	**0.120**	**0.040**	**0.011**	**1.127**	**1.043-1.218**
	Osteoporosis	IVW	13	0.179	0.073	**0.014**	**1.197**	**1.037–1.381**
		MR-Egger	13	0.520	0.130	**0.002**	**1.682**	**1.303–2.170**
		Weighted median	13	0.179	0.073	**0.030**	**1.197**	**1.037–1.381**
		MR-PRESSO	13	0.212	0.080	**0.008**	**1.236**	**1.056–1.447**
	Chronic periodontitis	IVW	13	0.193	0.079	**0.015**	**1.213**	**1.038–1.417**
		MR-Egger	13	0.379	0.179	0.057	1.461	1.030–2.073
		Weighted median	13	0.087	0.092	0.362	1.091	0.911–1.307
		MR-PRESSO	13	0.342	0.097	** < 0.001**	**1.408**	**1.164–1.703**
	Chronic kidney disease	IVW	12	0.176	0.054	**0.001**	**1.193**	**1.072–1.326**
		MR-Egger	12	0.270	0.125	0.056	1.310	1.025–1.675
		Weighted median	12	0.193	0.068	**0.005**	**1.213**	**1.061–1.387**
		MR-PRESSO	12	0.176	0.054	**0.008**	**1.193**	**1.072–1.326**

Bold values represent *P* < 0.05 for the result

*CI* confidence interval, *IVW* inverse variance weighted, *OR* odds ratio, *SE* standard error, *SNPs* single-nucleotide polymorphisms

Upon conducting a sensitivity analysis, heterogeneity was observed between studies of LUSC, osteoporosis and COPD as determined by Cochran’s Q statistics. Additionally, the MR-Egger intercept test revealed the presence of horizontal pleiotropy in the results of the analysis between COPD and osteoporosis. (Additional file 1: Table S6). For the leave-one-out analysis, our findings indicate that no individual genetic variant holds significant influence over the results (Additional file 1: Figure S1). Ultimately, due to the horizontal pleiotropy on the outcome involved between COPD and osteoporosis which cannot be adjusted by MR-PRSSO method, thus the preliminary result that liability to COPD could increase the risk of osteoporosis is unreliable.

## Reverse univariable MR analysis

**Fig. 3:**
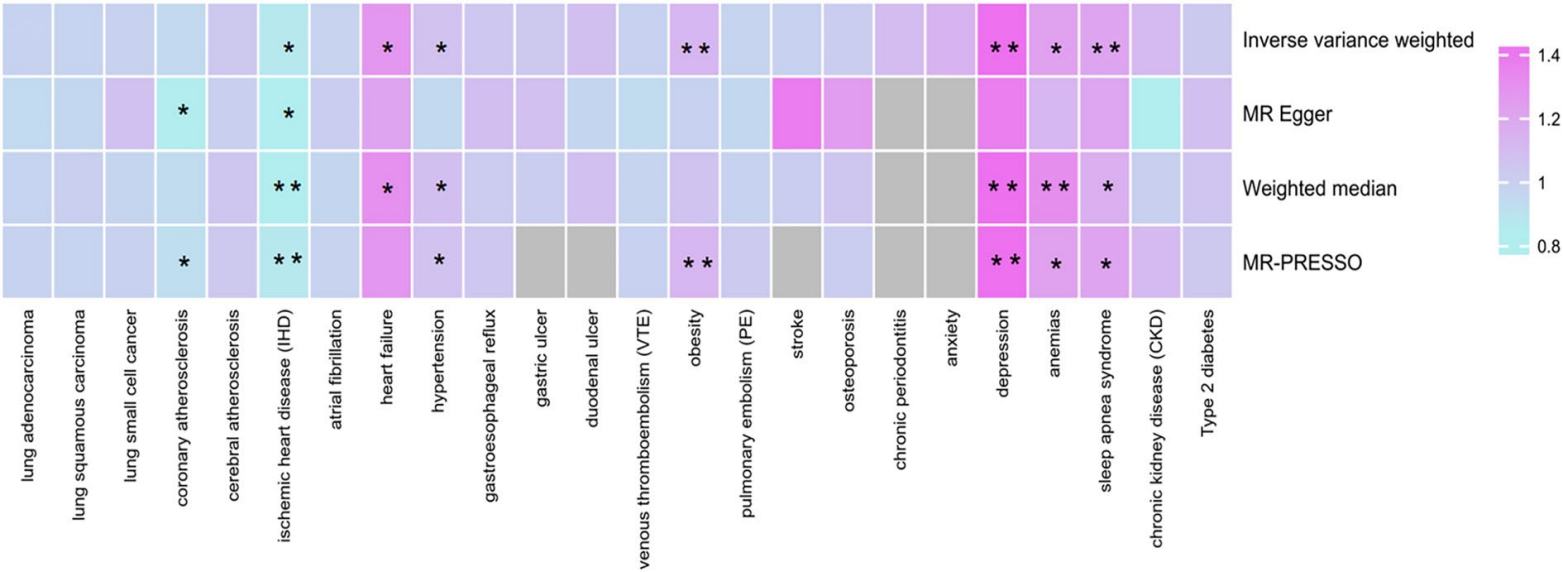
The preliminary MR assessments of the associations between genetic susceptibility to prevalent comorbidities and chronic obstructive pulmonary disease (COPD)

Preliminary reverse MR Analysis results suggest that genetic predisposition to specific comorbidities of COPD may also be associated with its outcome. From one perspective, genetic predisposition to IHD (IVW: OR = 0.867, 95% CI = 0.800–0.939, *P* < 0.001) may confer a protective effect on the development of COPD. Conversely, genetic susceptibility to HF (IVW: OR = 1.272, 95% CI = 1.084–1.493, *P* = 0.003), hypertension (IVW: OR = 1.067, 95% CI = 1.016–1.120, *P* = 0.010), obesity (IVW: OR = 1.128, 95% CI = 1.056–1.205, *P* < 0.001), depression (IVW: OR = 1.491, 95% CI = 1.257–1.770, *P* = 0.0005), anemias (IVW: OR = 1.227, 95% CI = 1.061–1.420, *P* = 0.006) and sleep apnea syndrome (IVW: OR = 1.209, 95% CI = 1.087–1.345, *P* < 0.001) may exert a detrimental effect on COPD (Fig. 3, Table 3 and Additional file 1: Table S7).

**Table 3:** The preliminary reverse UVMR results of significant relationship between COPD and its common comorbidities mainly based on IVW method (*P* < 0.05)

Exposure	Outcome	Method	nSNP	Beta	SE	*P*	OR	95% CI
Ischemic heart disease (IHD)	COPD	IVW	49	-0.143	0.041	** < 0.001**	**0.866**	**0.800–0.939**
		MR-Egger	49	-0.232	0.098	0.023	0.793	0.654–0.962
		Weighted median	49	-0.186	0.047	** < 0.001**	**0.830**	**0.757–0.911**
		MR-PRESSO	49	-0.143	0.041	**0.001**	**0.866**	**0.800–0.939**
Heart failure		IVW	4	0.241	0.082	**0.003**	**1.272**	1.084–1.493
		MR-Egger	4	0.190	0.332	0.625	1.210	0.631–2.320
		Weighted median	4	0.264	0.107	**0.014**	**1.302**	**1.055–1.607**
		MR-PRESSO	4	0.241	0.079	0.056	1.272	1.089–1.487
Hypertension		IVW	181	0.064	0.025	**0.010**	**1.067**	**1.016–1.120**
		MR-Egger	181	-0.058	0.075	0.438	0.944	0.816–1.092
		Weighted median	181	0.078	0.031	**0.011**	**1.082**	**1.018–1.149**
		MR-PRESSO	181	0.064	0.025	**0.011**	**1.067**	**1.016–1.120**
Obesity		IVW	29	0.120	0.034	** < 0.001**	**1.128**	**1.056–1.205**
		MR-Egger	29	-0.013	0.089	0.882	0.987	0.828–1.176
		Weighted median	29	0.061	0.042	0.148	1.063	0.979–1.154
		MR-PRESSO	29	0.120	0.034	**0.001**	**1.128**	**1.056–1.205**
Depression		IVW	14	0.400	0.087	** < 0.001**	**1.491**	**1.257–1.769**
		MR-Egger	14	0.317	0.519	0.552	1.373	0.497–3.795
		Weighted median	14	0.420	0.098	** < 0.001**	**1.523**	**1.257–1.844**
		MR-PRESSO	14	0.400	0.087	**0.001**	**1.491**	**1.257–1.769**
Anemias		IVW	7	0.205	0.074	**0.006**	**1.227**	**1.061–1.420**
		MR-Egger	7	0.118	0.146	0.455	1.125	0.846–1.497
		Weighted median	7	0.272	0.067	** < 0.001**	**1.313**	**1.152–1.496**
		MR-PRESSO	7	0.205	0.074	**0.033**	**1.227**	**1.061–1.420**
Sleep apnea syndrome		IVW	19	0.190	0.054	** < 0.001**	**1.209**	**1.087–1.345**
		MR-Egger	19	0.183	0.208	0.391	1.201	0.798–1.808
		Weighted median	19	0.150	0.076	0.048	1.162	1.001–1.348
		MR-PRESSO	19	0.190	0.054	**0.003**	**1.209**	**1.087–1.345**

Bold values represent *P* < 0.05 for the result

The sensitivity analysis revealed the presence of heterogeneity among the studies on IHD, hypertension, coronary atherosclerosis, anemias, depression, and COPD based on Cochran’s Q statistic. Furthermore, the MR-Egger intercept revealed the presence of horizontal pleiotropy in studies investigating the association among IHD, hypertension, coronary atherosclerosis, and COPD (Additional file 1: Table S8). No SNP was found to drive the above associations (Additional file 1: Figure S2). Finally, the outcomes that the association between IHD, hypertension, coronary atherosclerosis, anemias and COPD were unsteady due to the unadjustable horizontal pleiotropy.

## Multivariable MR analysis

Considering the close relationship between smoking and COPD, we employed the multivariable MR to adjust the effect of smoking confounding factors on the outcome. The results showed that after adjusting the influence of lifetime smoking index and age of initiation smoking confounders through MVMR, there was no causal relationship between COPD and chronic periodontitis. However, individuals who are liable to COPD could be prone to suffer LUSC (OR = 1.998, 95%CI = 1.249–3.197, *P* = 0.004), HF (OR = 1.174, 95%CI = 1.018–1.354, *P* = 0.027) and CKD (OR = 1.293, 95%CI = 1.070–1.564, *P* = 0.008) was consistent with the preliminary results based on MVMR-IVW. No significant horizontal pleiotropy was detected in the sensitivity analysis (Additional file 1: Table S9).

## The outcome of secondary analysis

The threshold of R^2^ for the selection of IVs was adjusted to 0.01 to investigate the potential impact of different IVs on the final outcome. Notably, our secondary MR analysis yielded consistent results with those obtained from the preliminary analysis (Additional file 1: Table S10–S14).

## Summary results of bidirectional MR analysis

**Fig. 4:**
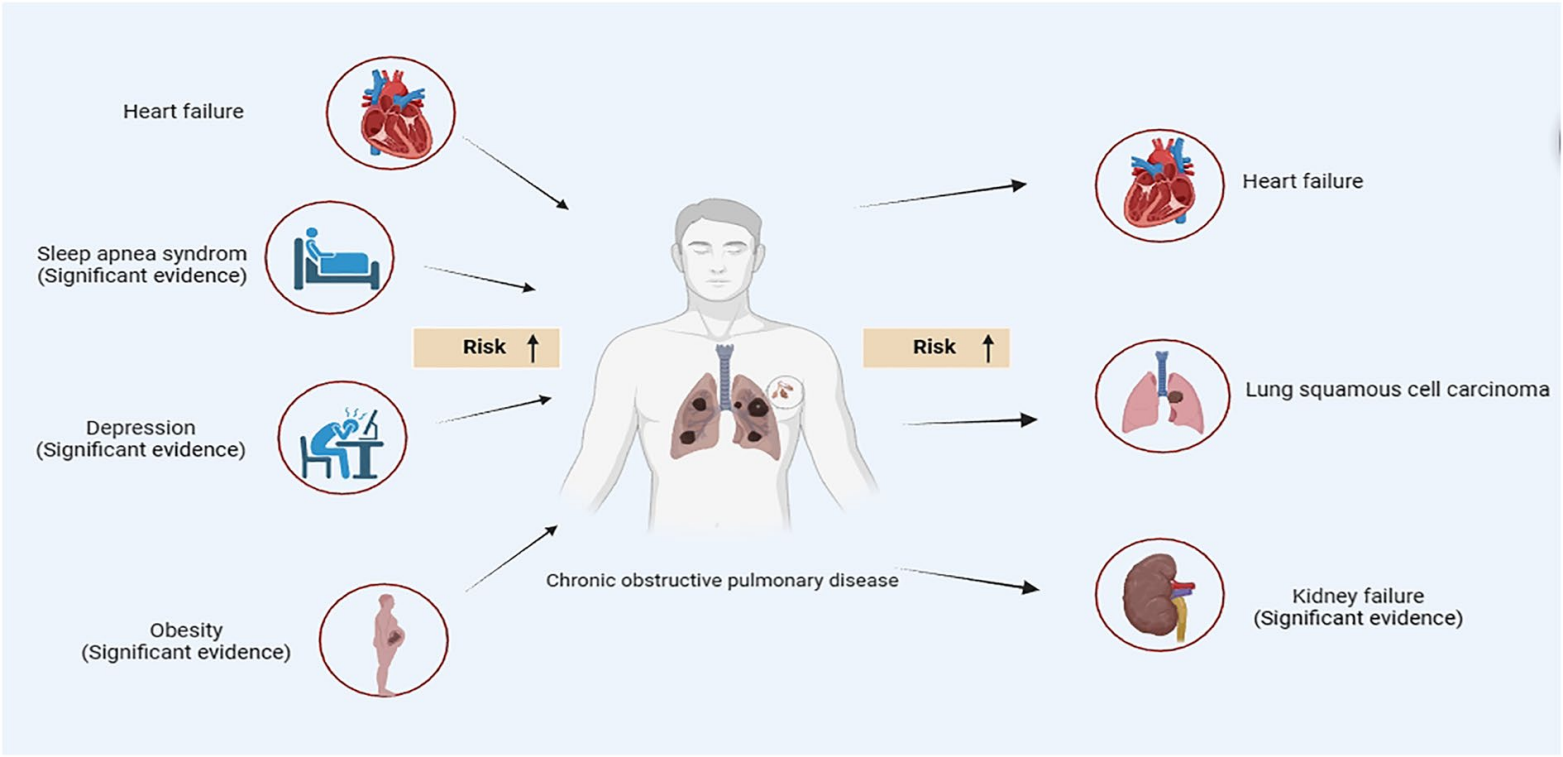
The overall outcome of our study

Collectively, after preliminary MR and subsequent sensitivity analysis, our study identified that liability to COPD may elevate the risk of LUSC, HF, and CKD. Additionally, our analysis revealed that genetic predisposition to HF, sleep apnea symptoms, depression, and obesity might also increase the susceptibility to COPD. Among them, there exists strong evidence linking CKD, sleep apnea syndrome, depression, obesity, and COPD. The comprehensive results of our research are depicted in Fig. 4.

## Discussion

To the best of our knowledge, the current study represented the first comprehensive MR analysis to investigate the causal relationship between COPD and its common comorbidities using the UVMR and MVMR approaches. Our findings revealed that genetic liability to COPD caused a higher risk of LUSC, HF and CKD. Additionally, genetic predisposition towards HF, obesity, sleep apnea syndrome, and depression increases the risk of COPD after excluding studies of existing horizontal pleiotropy, which could influence the final accuracy of our outcome.

Lung cancer, known for its rapid progression and poor prognosis, is a challenging disease to manage. Smoking is a shared risk factor for both COPD and lung cancer, interfering with investigating their causal relationship. However, Huang et al. [29] examined 24 pooled case–control studies from the International Lung Cancer Consortium (ILCCO). They demonstrated that 86% of the increased risk of SCLC occurs in individuals with COPD independent of smoking status. Besides, Wang et al. [30] reported that individuals diagnosed with COPD, specifically those with an emphysema-predominant phenotype, exhibited a heightened risk profile for SCLC. Several different hypotheses have been proposed to explain the possibility that COPD increases the risk of lung cancer. One possible explanation is that chronic inflammation and the accumulating release of free radicals may facilitate genetic malignant transformation during tissue reparation [31, 32]. Additionally, the accumulation of detrimental substances resulting from impaired pulmonary ventilation function and airway cilia destruction in patients with COPD could also contribute to the development of lung cancer [33]. Our comprehensive MR analysis indicated that individuals liable to COPD could have a higher chance of developing LUSC, but is not associated with lung adenocarcinoma and SCLC development, indicating that COPD may not increase the risk of all types of lung cancer. Therefore, additional experiments are necessary to elucidate the relationship between COPD and lung malignancy.

Our preliminary UVMR analysis showed that genetically predisposition to COPD could elevate the risk of periodontitis, but this finding was not statistically significant after correcting for smoking confounding factors by MVMR analysis, suggesting that smoking exposure is primarily a contributing factor to periodontitis rather than COPD itself. Although a comprehensive longitudinal cohort study conducted by Shen et al. [34] revealed that individuals with COPD had a 1.19 times greater likelihood of developing periodontitis compared to those without COPD, the study did not consider the additional effect of smoking on periodontitis.

Our research provided significant evidence that individuals with a genetic predisposition to COPD may be more susceptible to developing CKD. A comprehensive case-cohort study revealed that the likelihood of CKD in COPD patients was 1.61 times greater than in those without COPD [35]. Our study results present a contrasting view to those obtained from a previous MR Analysis, where reduced renal function was found to be associated with lower lung function. At the same time, the reciprocal was not statistically significant. This inconsistency could be attributed to the fact that the MR Analysis only examined indicators of pulmonary, forced expiratory volume in 1 s (FEV1)/forced vital capacity (FVC) and renal function, estimated glomerular filtration rate (eGFR) without considering the disease status [36]. The pathophysiological mechanisms underlying the development of CKD in patients with COPD are multifactorial and complex. However, previous studies have shown that the hypoxia and hypercapnia resulting from COPD can lead to renal vasoconstriction, thus compromising renal blood flow and sympathetic activation [37, 38]. Furthermore, the systematic inflammatory response triggered by COPD may also contribute to the development and progression of CKD.

It is worth noting that sleep apnea is a common condition that often coexists with COPD. The outcome of our reverse MR analysis provided significant evidence supporting the findings that individuals with a genetic predisposition for sleep apnea syndrome may be at an elevated risk of developing COPD. However, there have been varying findings between prior studies. Greenberg-Dotan et al. [39] discovered that sleep apnea can elevate the risk of COPD in a case–control study. In contrast, other community-based controlled studies of older adults have reported the opposite outcome [40]. The differences in the conclusions of the two studies may be due to the differences in the age and number of subjects enrolled in the study. Nonetheless, it has been well established that intermittent hypoxia-induced by sleep apnea syndrome can initiate the activation of hypoxia-driven and oxidative stress pathways, thereby triggering airway and systemic inflammatory responses [41]. To corroborate the results of our MR analysis, Additional file 1: studies with longer follow-up durations may be necessary.

Interestingly, our MR study revealed that genetic liability to depression could elevate the risk of developing COPD. A previous meta-analysis and systematic review, including sixteen relevant follow-up studies conducted by Atlantis et al. [42], demonstrated a link between depression and a higher risk of COPD outcomes. However, Martuccci [43] conducted a phenome-wide association study, which revealed no significant causal relationship between major depressive disorder and lung function at the genetic level, even after excluding the confounding factor of smoking. Nevertheless, it is essential to note that their study solely relied on a genome-wide association study (GWAS) of lung function, and their selection criteria exclusively encompassed patients with depression exhibiting severe symptoms. These methodological disparities may potentially contribute to discrepancies compared to our findings. The underlying mechanisms by which depression may contribute to the development of COPD remain unclear. However, several mechanisms have been speculated, such as depression leading to sympathetic activation and an increase in systemic inflammatory factors [27, 44]. Further studies are needed to establish the precise nature of the association between depression and COPD and the mechanisms that underlie this relationship.

Previous studies have established a significant relationship between COPD and obesity or Body Mass Index. Our latest analysis, which harnessed the power of the FinnGen database, corroborated these findings and suggested that obesity increases the susceptibility to COPD. The observed correlation may be attributed to the impact of obesity on chest volume, which can lead to physical repercussions. The inflammatory factors and hormonal changes associated with obesity are believed to exacerbate the progression of COPD [45, 46].

Our research has shown that liability to COPD are more likely to increase the risk of HF and vice versa. According to a prospective, multicenter, longitudinal cohort study conducted by LEKARZ et al. COPD was associated with a higher myocardial fibrosis burden and HF hospitalization compared to non-COPD patients [47]. Additionally, a recent experimental study has shown that elastase-induced COPD resulted in the development of diastolic cardiomyopathy in an animal model, independent of the confounding effects of cigarette smoke [48]. Given that HF is predominantly prevalent among elderly patients and the progression of COPD is a prolonged process, conducting a prospective cohort study to determine the causal relationship between HF and COPD would prove to be a formidable challenge. Notably, HF exists dysregulation of innate immunity and chronic inflammatory response. Furthermore, HF and COPD share common molecular and mechanistic pathways [49], which suggests a potential explanation for the increased vulnerability of HF patients to COPD development.

Our research has leveraged GWAS data as new as possible to scrutinize the causal connection between COPD and its common comorbidities. This approach has successfully addressed the challenge of establishing causality between diseases due to the impracticability of conducting a large-scale prospective study. Moreover, we have utilized various statistical techniques to ensure the precision of our outcomes and conducted a parallel sensitivity analysis to confirm the dependability of our findings.

Our study is subject to several limitations that warrant discussion. Firstly, to ensure homogeneity by race, we limited our study to populations of European ethnicity. Consequently, the generalizability of our findings is restricted to European populations, indicating the need for future studies that explore more diverse populations. Secondly, it is worth mentioning that majority of the study populations we analyzed were mainly derived from Finnish databases, which could potentially lead to some bias due to the inevitable overlap between exposure and outcome. In the future, the MR analysis based on GWAS databases from diverse sources is needed to verify our conclusions. Last but not least, although the MR analysis serves as an approach to infer the causal relationship between exposure and outcome using IVs, it is still crucial to confirm our findings with large-scale clinical studies or experiments.

## Conclusions

To sum up, our study has effectively established a causal connection between COPD and prevalent comorbidities using MR. This discovery could offer fresh insights into preventing and managing concerned diseases in clinical settings. Nonetheless, extensive prospective studies and animal experiments should be conducted to verify the precision and applicability of our results in the future.

### Supplementary Information

**Additional file 1:** **Figure S1.** Plots for "leave-one-out" analysis for the causal impact of COPD on potentially causal comorbidities. **Figure S2.** Plots for "leave-one-out" analysis for the causal impact ofpotentially causal comorbidities on COPD.

**Additional file 2: Table S1.** STROBE-MR checklist of Mendelian randomization study.** Table S2.** The comprehensive retrieved results of traits corresponding to SNPs in Penoscanncer V2 and GWAS catalog.** Table S3.** Comprehensive details of incorporated SNPs for each disease investigated (R^2^<0.001).** Table S4.** Sample overlap rate between exposure and outcome and potential bias.** Table S5.** The UVMR results of COPD to its frequently coexisting comorbidities (R^2^<0.001).** Table S6.** Pleiotropy and heterogeneity analysis of concerned forward UVMR analysis (R^2^<0.001).** Table S7.** The reverse UVMR results of COPD to its frequently coexisting comorbidities (R^2^<0.001).** Table S8.** Pleiotropy and heterogeneity analysis of concerned reverse UVMR analysis (R^2^<0.001).** Table S9.** The MVMR results with adjusting the smoking exposure (R^2^<0.001).** Table S10.** Comprehensive details of incorporated SNPs for each disease investigated (R^2^<0.01).** Table S11.** The UVMR results of COPD to its frequently coexisting comorbidities (R^2^<0.01).** Table S12.** Pleiotropy and heterogeneity analysis of concerned forward UVMR analysis (R^2^<0.01).** Table S13.** The reverse UVMR results of COPD to its frequently coexisting comorbidities (R^2^<0.01).** Table S14.** Pleiotropy and heterogeneity analysis of concerned reverse UVMR analysis (R^2^<0.01).

## Data Availability

The data used in this study were obtained from genome-wide association study summary statistics that were publicly released by genetic consortia. Interested parties may obtain the data by submitting a request to the corresponding author. Furthermore, all datasets generated for this study have been included in the article or additional files.

## Abbreviations

MR: Mendelian randomization
COPD: Chronic obstructive pulmonary disease
HF: Heart failure
LUSC: Lung squamous cell carcinoma
CKD: Chronic kidney disease
SCLC: Small cell lung cancer
IEU: Integrative Epidemiology Unit
SNP: Single nucleotide polymorphism
IVW: Inverse variance weighting
LD: Linkage disequilibrium
OR: Odds ratio
MR-PRESSO: MR pleiotropy residual sum and outlier
GWAS: Genome-wide association study
GERD: Gastroesophageal reflux disease
FEV1: Forced expiratory volume in 1 s
FVC: Forced vital capacity
eGFR: Estimated glomerular filtration rate
