# Supplementary material for: Unraveling the causality between chronic obstructive pulmonary disease and its common comorbidities using bidirectional Mendelian randomization
Source: Eur J Med Res. 2024 Feb 26;29:143. doi: 10.1186/s40001-024-01686-x (PMC10895842; doi:10.1186/s40001-024-01686-x)
Supplement: Supplementary file 1 — Additional file 1: Figure S1. Plots for "leave-one-out" analysis for the causal impact of COPD on potentially causal comorbidities. Figure S2. Plots for "leave-one-out" analysis for the causal impact ofpotentially causal comorbidities on COPD. [file 40001_2024_1686_MOESM1_ESM.pdf]

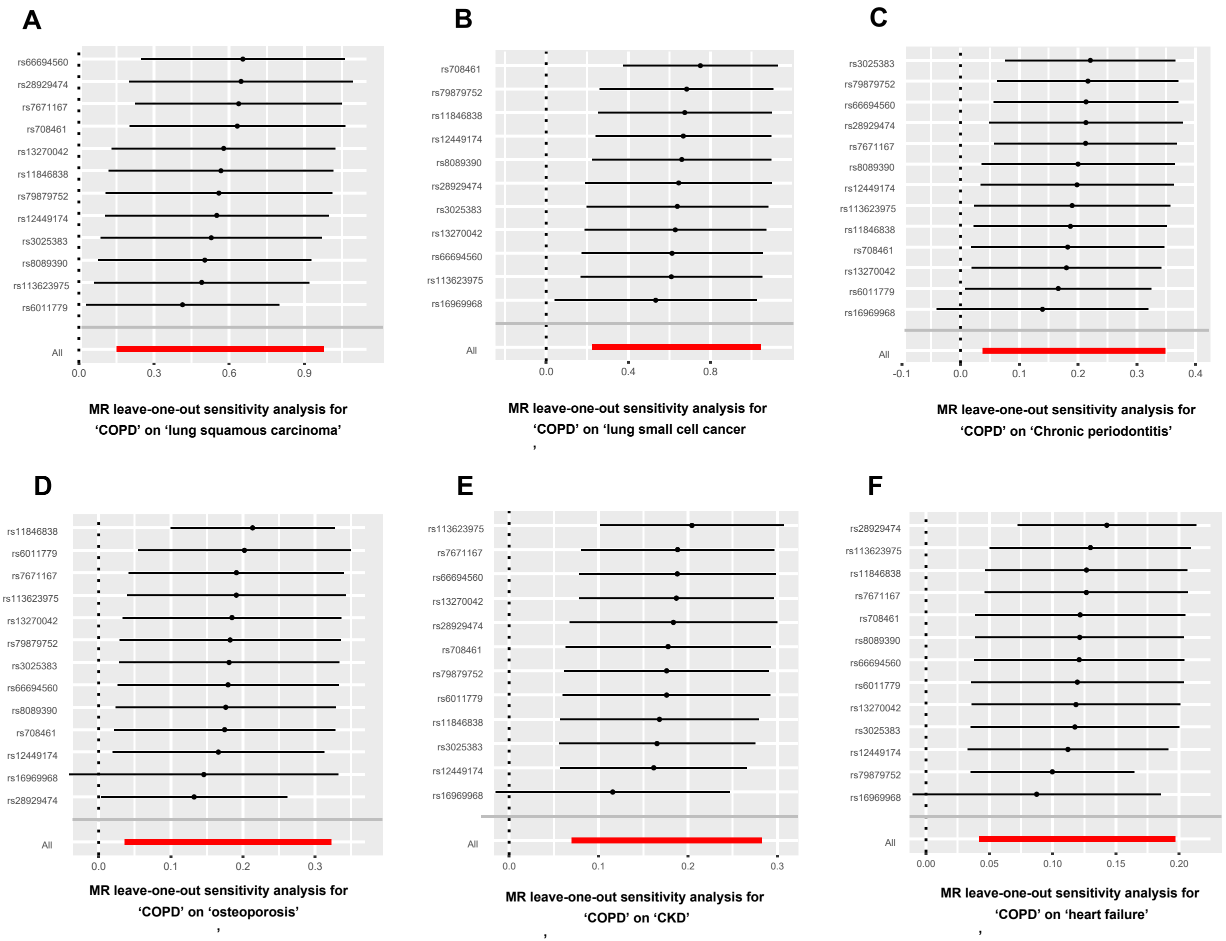

Supplementary Figure S1. Plots for "leave-one-out" analysis for causal impact of COPD on potentially causal comorbidities. Leave-one-out plot is a useful tool to assess whether the overall effect of the COPD on potentially causal commorbidities risk is influenced by any specific genetic variant. This method involves sequentially re-evaluating causal estimates after excluding one SNP at a time. The black points in the plot represent the effect estimates of the gut microbiota after omitting a particular SNP, and the black lines indicate the corresponding 95% confidence intervals. On the other hand, the red points in the plot denote the overall causal effect estimate of the gut microbiota on COPD risk using a set of SNPs, and the red lines represent the corresponding 95% confidence intervals.

A

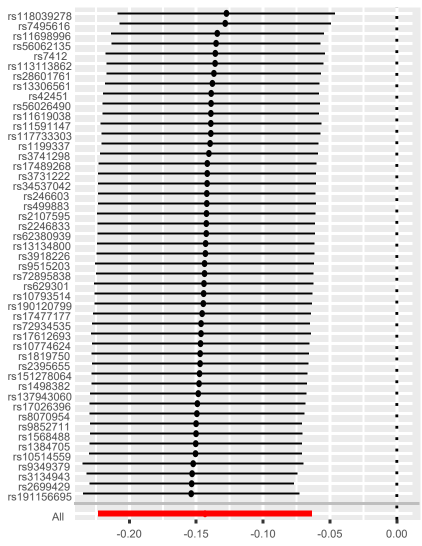

MR leave-one-out sensitivity analysis for
'IHD' on 'COPD'

B

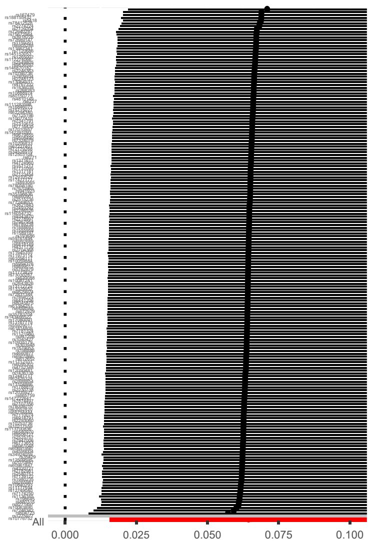

MR leave-one-out sensitivity analysis for
'hypertension' on 'COPD'

C

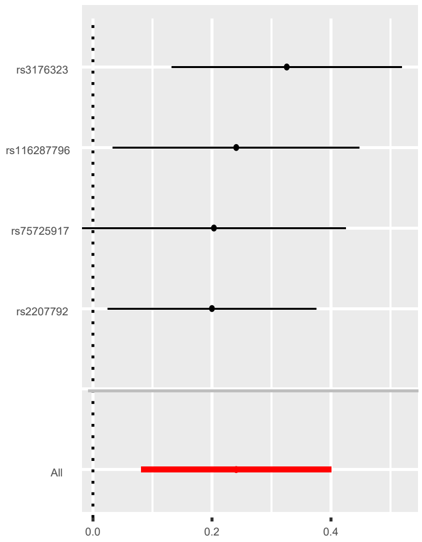

MR leave-one-out sensitivity analysis for
'heart failure' on 'COPD'

D

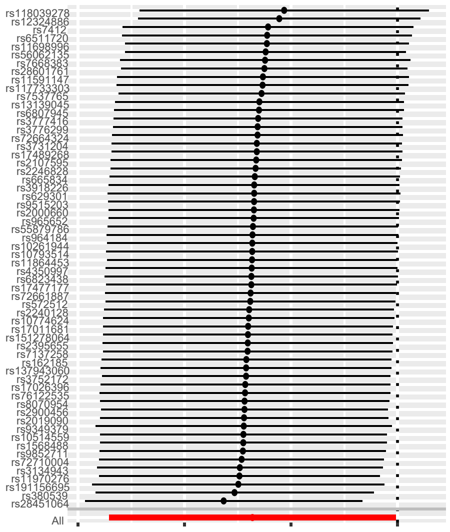

MR leave-one-out sensitivity analysis for
'coronary atherosclerosis' on 'COPD'

E

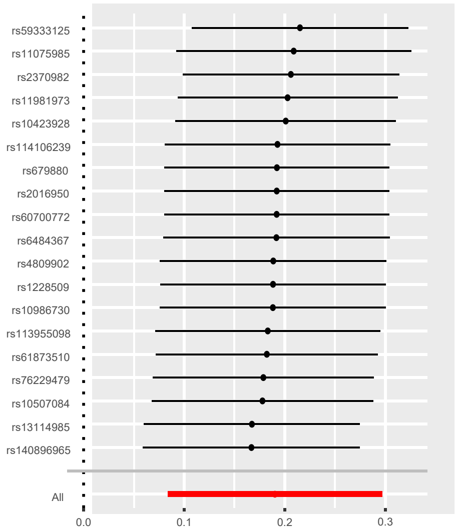

MR leave-one-out sensitivity analysis for
' sleep apnea syndrome' on 'COPD'

F

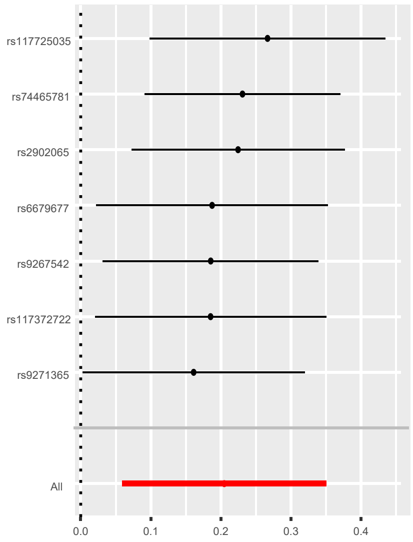

MR leave-one-out sensitivity analysis for
'anaemia' on 'COPD'

G

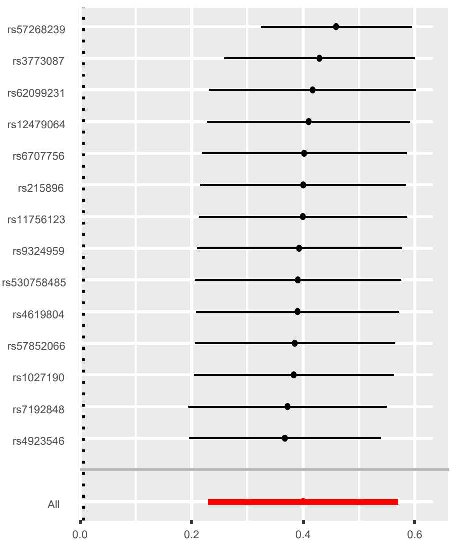

MR leave-one-out sensitivity analysis for
'depression' on 'COPD'

H

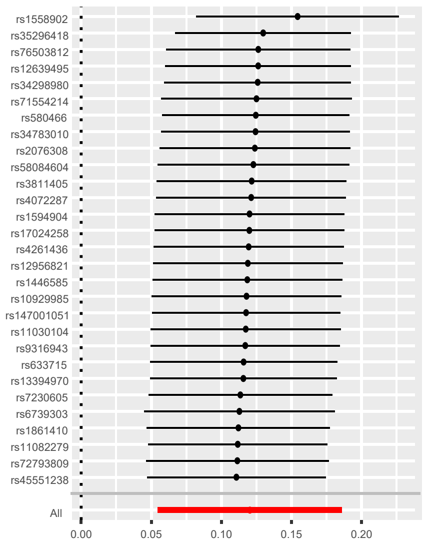

MR leave-one-out sensitivity analysis for
'obesity' on 'COPD'

Supplementary Figure S2. Plots for "leave-one-out" analysis for causal impact of potentially causal comorbidities on COPD.
